# A Novel Modification of Ureteral Reimplantation (Combined Technique) in Pediatric Patients: A Preliminary Case Series*

**DOI:** 10.5152/tud.2022.22107

**Published:** 2022-09-01

**Authors:** Deniz Demirci, Numan Baydilli, Emrah Kızılay

**Affiliations:** 1 Department of Pediatric Urology, Erciyes University Faculty of Medicine, Kayseri, Turkey

**Keywords:** Ureteral reimplantation, ureteral obstruction, vesicoureteral reflux

## Abstract

**Background:**

In this video, we present a new open ureteral reimplantation approach (combined technique) with preliminary results from 32 renal units.

**Material and methods:**

Written informed consent was obtained from the parents of the patients. We used a suprapubic Pfannenstiel incision to reach the bladder. After preparation of the ureters with the guidance of the vesicoureteral reflux surgery principle, they were moved from the bladder to the extravesical area. A submucosal tunnel was created above and below the old hiatus with reference to the old hiatus site. The required submucosal tunnel length is adjusted to be 2/3 above the old hiatus and 1/3 below the old hiatus. The ureters were carried down through the submucosal tunnel using a right-angle clamp and fixed to the bladder with 5/0 polyglactin sutures, step by step, respectively.

**Results:**

A total of 22 patients (9 females/13 males) with a median age of 7 (min: 2and max: 15) years were operated on using the combined technique. There were 16 cases with vesicoureteral reflux and 6 cases with unilateral obstructive megaureter. The success rate was found to be 100% for vesicoureteral reflux and 83.3% for primer obstructed megaureter. When we focus on the number of ureters, the overall success rate was found to be 97%.

**Conclusion:**

The vertical angulation or kinking of the ureter at the entrance to the bladder can be prevented in this modification. New orifice localization is close to the normal position. Short tunnel length is out of the question in this modification. We think that with potential surgical advantages, a novel combined technique can be used in ureteral reimplantation.

## Introduction

Various intravesical or extravesical surgical techniques have been described for ureteral reimplantation. Although these techniques have been widely used, there are some postoperative limitations such as localization difficulty of new ureteral orifices, obstructive kinking, high reflux recurrence rate, or voiding problems for Cohen, Politano, Glenn Anderson, Lich–Gregoir, respectively. To overcome these postoperative complications, we present a new open ureteral reimplantation approach (combined technique) with preliminary results of 22 cases and 32 renal units.

## Materials and Methods

After informed consent was obtained from the parents of the participants before the operation, we used a suprapubic Pfannenstiel incision to reach the bladder under general anesthesia. After preparation of the ureters with the guidance of the vesicoureteral reflux surgery principle, they were moved from the bladder to the extravesical area. A submucosal tunnel was created above and below the old hiatus with reference to the old hiatus site. The required submucosal tunnel length is adjusted to be 2/3 above the old hiatus and 1/3 below the old hiatus. The ureters were carried down through the submucosal tunnel using a right-angle clamp by gentle maneuvers. Ureters were fixed to the bladder with 5/0 polyglactin sutures, step by step, respectively. A J stent was placed into the ureter on both sides. The urethral catheter was removed on the seventh postoperative day. The J stents were removed 4 weeks after the operation. The efficacy of the surgery was checked with ultrasonography (USG) and voiding cystourethrography (VSUG) in the first month after the J stent was removed. In the first year of follow-up, urinalysis, urine culture, and USG were used every 3 months.

## Results

A total of 22 patients (9 females /13 males) with a median age of 7 (min: 2 and max: 15) years were operated on using the combined technique. There were 16 cases with vesicoureteral reflux and 6 cases with unilateral primer obstructive megaureter. The median operative time was 80 (70-95) minutes with minimal blood loss. The median length of hospital stay was 6 (min: 4 and max: 8) days. Reflux and/or ureteral/pelvicalyceal dilatation were not observed in 21 (95.5%) cases. Grade I vesicoureteral reflux (VUR) was detected in VSUG in the postoperative period of 1 (4.5%) patient who previously had unilateral ureterostomy due to unilateral primer obstructive megaureter (Table 1). However, this reflux completely disappeared from the upper urinary system at the end of the voiding phase. The success rate was found to be 16/16 (100%) for VUR and 5/6 (83.3%) for obstructed megaureter. When we focus on the number of ureters, the overall success rate was found to be 97% (31/32). Febrile urinary tract infection (UTI) was not detected in any of the patients who were followed up for a median of 10 (min: 6 and max: 48) months (Table 1).

## Discussion

Ureteral reimplantation is generally used for surgical correction of VUR or obstructed megaureter, and various techniques have been described. Reimplantation of the ureter with an adequate length of the submucosal tunnel is common in all described methods. The Cohen technique is one of the most popular and reliable procedures with a wide range of applications and simplicity. Nevertheless, the major disadvantage of this method is certainly the displacement of the ureteral orifice to the contralateral side, which may make further endoscopic procedures through the ureter more difficult.^1^ The Glenn–Anderson and Politano–Leadbetter techniques are both intravesical procedures and do not significantly alter the normal anatomical alignment of the ureter.^2^ The disadvantage of Glenn–Anderson technique is a relatively short length of the submucosal tunnel and sufficient tunnel length cannot be achieved in the case of the severely dilated ureter or in pediatric patients with small capacity bladder.^3^ Although Politano–Leadbetter technique is a safe and standardized procedure with an acceptable complication rate, injuries to the intestine and the ductus deferens can occur, and there is an additional risk of ureteral kinking at the new hiatus with subsequent obstruction. Therefore, some modifications had been reported for Politano-Leadbetter, but the ureteral kinking problem still exists .^2,4^

Extravesical ureteral reimplantation (Lich–Gregoir) can lead to neurological damage and subsequent bladder-emptying problems. Although this complication is transient and reversible, the association with postoperative urinary retention has limited its use in treating bilateral cases.^2^ ^5^ Previously published surgical illustrations were used as a template for the video article.^4,6^

In the current study, to overcome postoperative disadvantages in all ureteral reimplantation techniques, we presented a novel combined approach (combination of modified Politano–Leadbetter and Glenn–Anderson technique). This technique demonstrated a success rate of 100% for the correction of VUR, 83% for the treatment of obstructive megaureter, and 97% for all ureteral reimplantation.

The relatively low patient population can be considered a limitation of the study. However, the success rate is high, the complication rate is low, and the results regarding the technique will be further refined by increasing the number of cases and the variety of cases.

In conclusion, the vertical angulation or kinking of the ureter at the entrance to the bladder can be prevented in this modification. The new orifice localization may be useful for surgical instrumentation to ureteral access in later life. Short tunnel length is out of the question in this modification. Therefore, it can provide adequate submucosal length for all cases. We think that with potential surgical advantages, a novel combined technique can be used in ureteral reimplantation.

## Figures and Tables

**Table 1.** Patient Characteristics

Patient Characteristics	n = 22 Patients (32 Renal Units)
**Age (years), **median (min-max)	7.2 (2-15)
**Gender, n (%)**	
Male/female	13 (59) / 9 (41)
**Reason for surgery, n (%)**	
VUR/obstructive megaureter	16 (72.8) / 6 (27.2)
**VUR, n (%)**	
Unilateral/bilateral	6 (37.5) / 10 (62.5)
**VUR grade, n (%)**	
Grade III	7 (43.7)
Grade IV	7 (43.7)
Grade V	2 (12.6)
**Obstructive megaureter, n (%)**	
Unilateral/bilateral	6 (100) / 0 (0)
**Complication, n (%)**	
Reflux recurrence (grade I = in 1 case with obstructive megaureter)	1 (4.5)
Ureteral stricture, bowel injury, perivesical hematoma	0 (0)
**Success for renal units, n (%)**	
VUR	26 /26 (100)
Obstructive megaureter	5/6 (83.3)
Overall (by the number of ureters)	31/32 (97)

VUR, vesicoureteral reflux.
